# An actin-binding protein ESPN is an independent prognosticator and regulates cell growth for esophageal squamous cell carcinoma

**DOI:** 10.1186/s12935-018-0713-x

**Published:** 2018-12-29

**Authors:** Shau-Hsuan Li, Hung-I Lu, Wan-Ting Huang, Yen-Hao Chen, Chien-Ming Lo, Ya-Chun Lan, Wei-Che Lin, Hsin-Ting Tsai, Chang-Han Chen

**Affiliations:** 1 Department of Hematology-Oncology, Kaohsiung Chang Gung Memorial Hospital and Chang Gung University College of Medicine, Kaohsiung, Taiwan, ROC; 2 Department of Thoracic & Cardiovascular Surgery, Kaohsiung Chang Gung Memorial Hospital and Chang Gung University College of Medicine, Kaohsiung, Taiwan, ROC; 3 Department of Pathology, Kaohsiung Chang Gung Memorial Hospital and Chang Gung University College of Medicine, Kaohsiung, Taiwan, ROC; 4 Department of Diagnostic Radiology, Kaohsiung Chang Gung Memorial Hospital and Chang Gung University College of Medicine, Kaohsiung, Taiwan, ROC; 5 Department of Applied Chemistry, and Graduate Institute of Biomedicine and Biomedical Technology, National Chi Nan University, Nantou, 54561 Taiwan, ROC; 6 Guangdong Institute of Gastroenterology, and Guangdong Provincial Key Laboratory of Colorectal and Pelvic Floor Disease, Sun Yat-sen University, No. 26 Yuanchun Er Heng Road, Guangzhou, 510020 Guangdong China

**Keywords:** Esophageal cancer, Squamous cell carcinoma, ESPN

## Abstract

**Background:**

ESPN (Espin), an actin filament-binding protein, plays an important role in regulating the organization, dimensions, dynamics, and signaling capacities of the actin filament-rich, microvillus-type specializations that mediate sensory transduction in various mechanosensory and chemosensory cells. Recent few studies show that ESPN regulates metastasis and cell proliferation in melanoma. However, the significance of ESPN in other cancers such as esophageal squamous cell carcinoma (ESCC) remains largely unknown.

**Methods:**

Immunohistochemistry was performed in 169 patients with ESCC and correlated with clinicopathological features and survival. The functional role of ESPN in ESCC cells was determined by ESPN-mediated siRNA.

**Results:**

Univariate analyses showed that high ESPN expression was associated with inferior overall survival (P = 0.005) and disease-free survival (P = 0.035). High ESPN expression was an independent prognosticator in multivariate analysis for overall survival (P = 0.009, hazard ratio = 1.688) and disease-free survival (P = 0.049, hazard ratio = 1.451). The 5-year overall survival rates were 30% and 54% in patients with high and low expression of ESPN, respectively. Inhibition of endogenous ESPN in ESCC cells decreased ESCC growth by reducing cell proliferating rates.

**Conclusions:**

High ESPN expression is independently associated with poor prognosis in patients with ESCC and downregulation of ESPN inhibits ESCC cell growth. Our results suggest that ESPN may be a novel therapeutic target for patients with ESCC.

## Background

Esophageal cancer occurs worldwide. The incidence and mortality of esophageal cancer rank ninth and sixth among all malignancies in the world, respectively [1]. The major two histologic types of esophageal cancer are squamous cell carcinoma and adenocarcinoma. In contrast to adenocarcinoma arising from Barrett’s esophagus in Western countries, the major disease phenotype in the Asia–Pacific region is esophageal squamous cell carcinoma (ESCC) [2, 3]. Despite improvements in surgical techniques and perioperative management, and surgery combined with chemotherapy and radiotherapy, patients still have recurrences after curative treatment. Thus, the prognosis of patients with ESCC is unsatisfactory [2, 4, 5]. Therefore, identification of biomarker to predict ESCC prognosis could improve risk-adapted treatment approaches and may offer further insight that benefit the development of novel therapeutic target.

ESPN (Espin), a molecular mass of approximately 110 kDa, is found to be specific to testis among the rat organs [6]. ESPN is localized to the parallel actin bundles in the junctional plaque of Sertoli cell ectoplasmic specializations. ESPN controls the formation and extension of protrusion structure by interacting with F-actin to modulate F-actin cytoskeleton remodeling in cells [7]. *ESPN* gene mutation causes hereditary deafness and vestibular dysfunction accompanied by stereociliary shortening in jerker mice and humans [8, 9]. In human cancers, ESPN plays a growth and metastatic regulator for melanoma [10, 11]. Enhanced ESPN expression in melanoma accelerates tumor growth, migration, and invasion. In contrast, suppression endogenous of ESPN significantly impairs the malignant phenotypes of melanoma cancer cells. Mechanistically, ESPN inhibition in melanoma promotes p21 and p27 protein expressions and arrests cell cycle at G1 phase. This result demonstrated that ESPN might play an oncogene and cell cycle regulator in melanoma [10]. To our knowledge, however, the expression and function of ESPN in ESCC is still elusive.

In the current study, we determine the expression and clinical relevance of ESPN in ESCC and uncover its biological role in ESCC cells.

## Materials and methods

### Patient population

We retrospectively reviewed ESCC patients treated with esophagectomy at Kaohsiung Chang Gung Memorial Hospital. This retrospective study was approved by Chang Gung Medical Foundation Institutional Review Board. Patients receiving preoperative chemoradiotherapy, preoperative chemotherapy, or preoperative radiotherapy, and patients with synchronous cancers in the other organ were excluded. Finally, 169 patients were identified. Patients undergoing surgery had a radical esophagectomy with cervical esophagogastric anastomosis (McKeown procedure) or an Ivor Lewis esophagectomy with intrathoracic anastomosis, reconstruction of the digestive tract with gastric tube, and pylorus drainage procedures. All patients received two-field lymph node dissection. The 7th American Joint Committee on Cancer (AJCC) staging system was applied to determine the pathological TNM stage [12]. Overall survival (OS) was calculated from the time of surgery to death as a result of all causes. Disease-free survival (DFS) was calculated from the time of surgery to the recurrence or death from any cause without evidence of recurrence.

### Immunohistochemistry

Immunohistochemistry staining was performed using an immunoperoxidase technique. Staining was performed on slides (4 μm) of formalin-fixed, paraffin-embedded tissue sections with primary antibodies against ESPN (N-term, 1:50, GeneTex, Inc. Irvine, CA, USA). Briefly, after deparaffinization and rehydration, the retrieval of the antigen was performed by treating the slides in 10 mmol/L citrate buffer (pH 6.0) in a hot water bath (95 °C) for 20 min. Endogenous peroxidase activity was blocked for 15 min in 0.3% hydrogen peroxide. After blocking with 1% goat serum for 1 h at room temperature, the sections were incubated with primary antibodies for at least 18 h at 4 °C overnight. Immunodetection was performed using the LSAB2 kit (Dako, Carpinteria, Calif) followed by 3–3′-diaminobenzidine for color development and hematoxylin for counterstaining. Incubation without the primary antibody was used as a negative control, and normal testis tissue was used as a positive control for ESPN. The staining was assessed by 2 pathologists (S.L.W. and W.T.H) without any information about clinicopathologic features or treatment outcome. A semi-quantitative immunoreactive score (IRS) was used to evaluate the immunohistochemistry staining [13]. The IRS was calculated by multiplying the staining intensity (graded as: 0 = no staining, 1 = weak staining, 2 = moderate staining, and 3 = strong staining) and the percentage of positively stained cells (0 = no stained cell, 1 ≤ 10% of stained cells, 2 = 10–50% of stained cells, 3 = 51–80% of stained cells, and 4  ≥ 80% of stained cells). The criterion for positive staining was a specimen with an IRS ≥ 6.

### Cell line and culture conditions

Human ESCC cell lines KYSE270 and TE10 were purchased from the Public Health England (PHE, London, UK) and RIKEN BioResource Research Center (RBRC, Ibaraki, Japan). Cells were grown in RPMI (Roswell Park Memorial Institute) 1640 medium supplemented with 10% fetal calf serum, and 2 mM glutamine and were maintained at 37 °C in a 5% CO2 air humidified incubator. All cell lines were regularly authenticated and tested for absence of mycoplasma.

### Cell transfection and siRNA

All ESCC cells were seeded in a 6-well plate at a density of 5 × 10^5^ for 24 h before transfection. Transient transfection was performed using Lipofectamine 2000 (Invitrogen) according to the manufacturer’s instructions, and the cells were subjected to immunoblotting and Q-RT-PCR at 48 h after the transfection. The two ESPN siRNAs and Silencer Negative control (AM4611) were designed and synthesized by Thermo Fisher Scientific (Taipei, Taiwan). Two ESPN siRNA sequences were 5′-CCGCAGAGCUGGAGGCUAA-3′ and 5′-GGACGCUGGGCUACGAUGA-3′.

### RNA extraction and Q-RT-PCR

Total RNA was extracted from cell lines by the TRIzol reagent and 2 μg of total RNA was reverse-transcribed to cDNA using M-MLV (Moloney murine leukaemia virus) Reverse Transcriptase (Life Technologies) with oligo(dT)20 primers. For Q-RT-PCR, diluted cDNA samples were amplified by QuantiFast SYBR Green PCR kit (Qiagen) using specific primers. Each sample was assayed in triplicate and gene expression was normalized with GAPDH expression.

### Western blotting

Total proteins extracted from cell lines were lysed with NP-40 lysis buffer (1% NP-40, 0.5% sodium deoxycholate, 150 mM NaCl and 50 mM Tris–HCl, pH 7.5, with protease inhibitor cocktails). The supernatants from cell lysates were collected for separation by SDS–polyacrylamide gel electrophoresis and transferred to PVDF membrane. Antibody against ESPN (GTX81675; GeneTex, Irvine, U.S.A), p21 (#2947, Cell signaling technology), p27 (#3686, Cell signaling technology) or β-actin (sc-130656; Santa Cruz Biotechnology) were used. After washing, the membrane was incubated with secondary antibodies conjugated to HRP for 1 h at room temperature. The signal was detected using ECL Western Detection Reagent.

### Cell growth assay and colony formation assay

The viability of the transfected cells was estimated at indicated time points (on 24, 48 and 72 h) after seeding into 96-well culture plates at the density of 3000 cells/well. 20 µl of MTT (3-(4, 5-dimethyl-2-thiazolyl)-2, 5-diphenyl-2-H-tetrazolium bromide) reagent (5 mg/ml) was added into each well, and incubated for 4 h. After discarding the supernatant, 100 µl DMSO was added to dissolve the formazan product. The optical density (OD) value was measured by a microplate reader at 490 nm. For colony formation assay, cells were inoculated in six-well plates, at a density of 300 cells/well and incubated for 8 days at 37  °C. Then cells were stained with 1% crystal violet for 30 min. The plates were washed with PBS and dried before microscopic evaluation.

#### Flow cytometry for cell cycle analysis

siESPN transfectants in KYSE270 and TE10 were harvested, fixed in 1% paraformaldehyde, and stained with PI (5 mg/ml) in PBS supplemented with RNase A for 30 min at room temperature. The cell cycle distribution were determined using Flow cytometer and Flow Jo software.

### Statistical analysis

For patient data, statistical analysis was performed using the SPSS 17 software package. The Chi square test and Fisher’s exact test were used to compare data between the two groups. For survival analysis, the Kaplan–Meier method was used for univariate analysis, and the difference between survival curves was tested by a log-rank test. Significant parameters at univariate level were entered into Cox regression model to analyze their relative prognostic importance. For cell line experiments, statistical analysis was performed using GraphPad Prism 5.0. Measurement data are presented as mean ± standard deviation (SD). Statistical analyses were performed with Student’s t test (two-tailed) and one-way ANOVA as appropriate. For all analyses, a P value < 0.05 was considered statistically significant.

## Results

### Patient characteristics

A total of 169 patients with ESCC receiving surgery were collected in this study with a median age of 55 years (range 29–80 years). The characteristics of 169 patients were summarized in Table 1. Among them, 163 (96%) were men and 6 (4%) were women. Under T classification, 56 (33%) patients were T1; 34 (20%) were T2; 64 (38%) were T3; and 15 (9%) were T4. Furthermore, under N classification, 115 (68%) patients were N0; 33 (20%) were N1; 14 (8%) were N2; and 7 (4%) were N3. The 7th AJCC stages were stage IA in 6 (4%) patients, stage IB in 46 (27%) patients, stage IIA in 26 (15%) patients, stage IIB in 45 (27%) patients, stage IIIA in 17 (10%) patients, stage IIIB in 7 (4%) patients, and stage IIIC in 22 (13%) patients. Further analyses of histologic grades showed a grade 1 lesion in 15 (9%) patients, grade 2 in 110 (65%) patients, and grade 3 in 44 (26%) patients. Primary tumor location was found to be upper in 27 (16%) patients, middle in 63 (37%) patients, and lower in 79 (47%) patients. Of these 169 patients, resection margins were positive for residual tumor in 19 (11%) patients. At the time of analysis, the median periods of follow-up were 67 months (range 60–238 months) for the 60 survivors and 34 months (range 1–238 months) for all 169 patients. The 5-year overall and disease-free survival rates of these 169 patients were 42% and 36%.

**Table 1:** Characteristics of 169 patients with ESCC receiving esophagectomy

Age	
Median	55
Mean	56.3
Range	29–80
Sex	
Male	163 (96%)
Female	6 (4%)
Primary tumor location	
Upper	27 (16%)
Middle	63 (37%)
Lower	79 (47%)
Pathological T classification	
T1	56 (33%)
T2	34 (20%)
T3	64 (38%)
T4	15 (9%)
Pathological N classification	
N0	115 (68%)
N1	33 (20%)
N2	14 (8%)
N3	7 (4%)
Pathological 7th AJCC Stage	
IA	6 (4%)
IB	46 (27%)
IIA	26 (15%)
IIB	45 (27%)
IIIA	17 (10%)
IIIB	7 (4%)
IIIC	22 (13%)
Histological grading	
Grade 1	15 (9%)
Grade 2	110 (65%)
Grade 3	44 (26%)
Surgical margin	
Negative	150 (89%)
Positive	19 (11%)
ESPN expression	
Low expression	83 (49%)
High expression	86 (51%)

*AJCC* American Joint Committee on Cancer

### Correlation between clinicopathologic parameters and ESPN expression

**Fig. 1:**
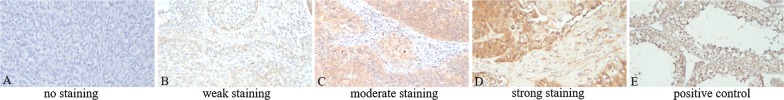
Immunohistochemical staining of ESPN in esophageal squamous cell carcinoma. **A** Representative example of ESPN expression with no staining. Original magnification, ×200. **B** Representative example of ESPN expression with weak staining. Original magnification, ×200. **C** Representative example of ESPN expression with moderate staining. Original magnification, ×200. **D** Representative example of ESPN expression with strong staining. Original magnification, ×200. **E** ESPN immunoreactivity was present in cells in seminiferous ducts in human testis used as a positive control. Original magnification, ×200

Among the 169 patients collected, high ESPN expression was identified in 86 (51%) patients (Fig. 1). The associations between clinicopathological parameters and ESPN expression were summarized in Table 2. We did not observe any association between ESPN expression with any clinicopathologic parameters including age, primary tumor location, histologic grading, T classification, N classification, and 7th AJCC Stage.

**Table 2:** Associations between ESPN expression and clinicopathological parameters in 169 patients with ESCC receiving esophagectomy

Parameters	ESPN expression		
	Low	High	P value
Age			
< 55 y/o	42	41	0.70
≥ 55 y/o	41	45	
Sex			
Male	81	82	0.68
Female	2	4	
Primary tumor location			
U/M	41	49	0.32
L	42	37	
Pathological T classification			
T1/T2	49	41	0.14
T3/T4	34	45	
Pathological N classification			
N0	59	56	0.41
N1/2/3	24	30	
Pathological 7th AJCC Stage			
I/II	64	59	0.21
III	19	27	
Histological grading			
Grade 1/2	60	65	0.63
Grade 3	23	21	
Surgical margin			
Negative	72	78	0.42
Positive	11	8	

*AJCC* American Joint Committee on Cancer

### Survival analyses

**Fig. 2:**
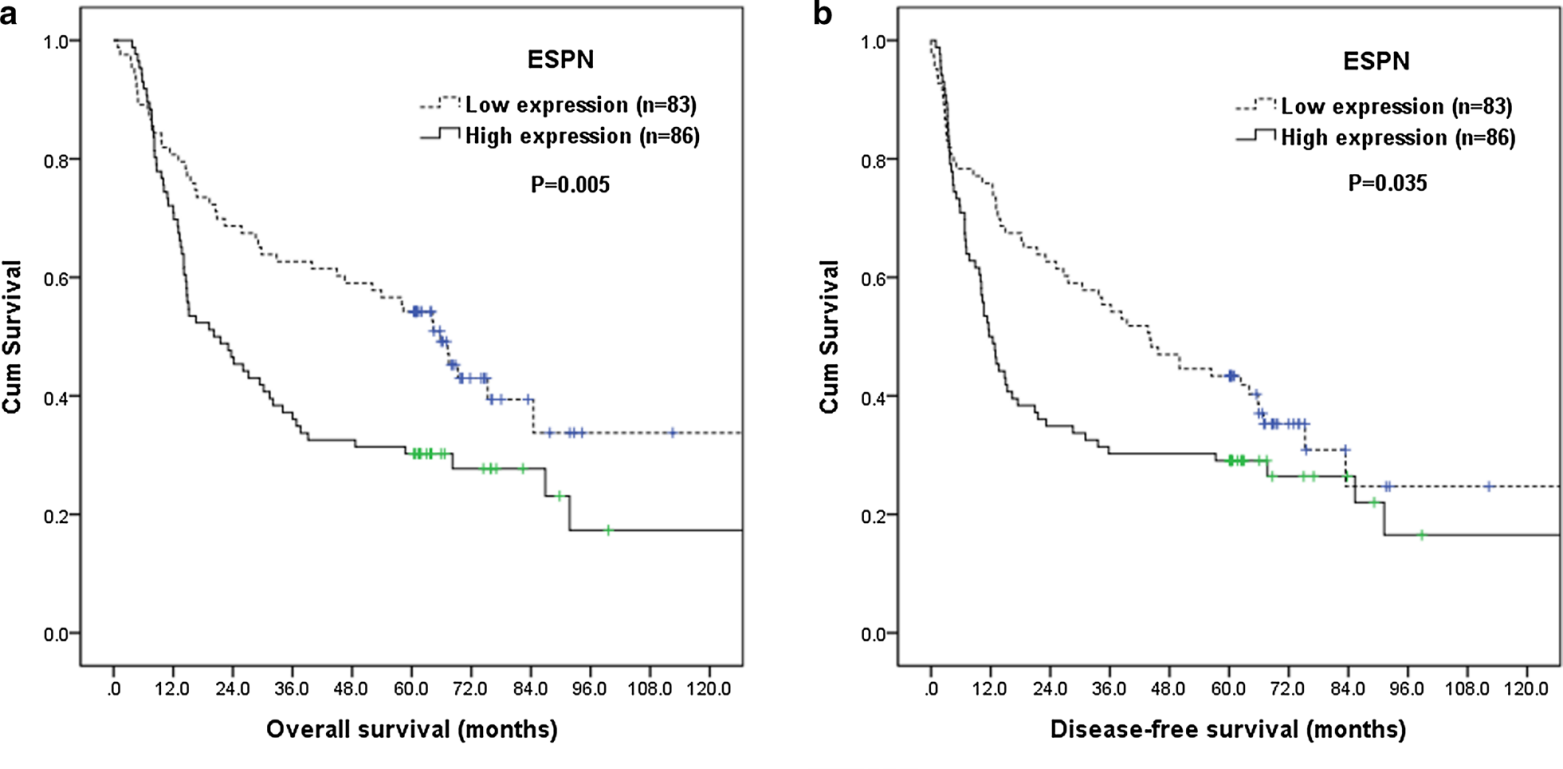
Kaplan–Meier curves according to ESPN status. **a** Overall survival according to ESPN status. **b** Disease-free survival according to ESPN status

The correlations of patients’ survival with clinicopathological parameters and ESPN expression were summarized in Table 3. By log-rank tests, 7th AJCC stage III (P < 0.001), T classification, T3/4 (P < 0.001), N classification, N1/2/3 (P < 0.001), positive surgical margin (P = 0.01), and high ESPN expression (P = 0.005, Fig. 2a) were associated with inferior OS. Furthermore, 7th AJCC stage III (P < 0.001), T classification, T3/4 (P < 0.001), N classification, N1/2/3 (P < 0.001), positive surgical margin (P = 0.02), and high ESPN expression (P = 0.035, Fig. 2b) were associated with worse DFS.

**Table 3:** Results of univariate log-rank analysis of prognostic factors for overall survival and disease-free survival in 169 patients with ESCC receiving esophagectomy

Factors	No. of patients	Overall survival (OS)		Disease-free survival (DFS)	
		5-year OS rate (%)	P value	5-year DFS rate (%)	P value
Age					
< 55 y/o	83	48	0.35	43	0.15
≥ 55 y/o	86	36		29	
Location					
U/M	90	43	0.98	37	0.70
L	79	41		36	
Pathological T classification					
T1/2	90	58	< 0.001*	48	< 0.001*
T3/4	79	24		23	
Pathological N classification					
N0	115	53	< 0.001*	45	< 0.001*
N1/2/3	54	19		17	
Pathological 7th AJCC stage					
I/II	123	52	< 0.001*	44	< 0.001*
III	46	15		15	
Histological grading					
Grade 1/2	125	46	0.088	38	0.18
Grade 3	44	32		30	
Surgical margin					
Negative	150	45	0.01*	38	0.02*
Positive	19	21		21	
ESPN expression					
Low expression	83	54	0.005*	43	0.035*
High expression	86	30		29	

*AJCC* American Joint Committee on Cancer

* Statistically significant

In multivariate analysis, high ESPN expression (P = 0.009, hazard ratio = 1.688, 95% confidence interval 1.141–2.498) remained independently associated with inferior OS, together with T classification, T3/4 (P = 0.005, hazard ratio = 2.033, 95% confidence interval 1.242–3.327) and N classification, N1/2/3 (P = 0.005, hazard ratio = 2.261, 95% confidence interval 1.281–3.992). For DFS, high ESPN expression (P = 0.049, hazard ratio = 1.451, 95% confidence interval 1.002–2.100), T classification, T3/4 (P = 0.005, hazard ratio = 1.932, 95% confidence interval 1.215–3.073), and N classification, N1/2/3 (P = 0.007, hazard ratio = 2.096, 95% confidence interval 1.225–3.586) represented an independent adverse prognosticator. The 5-year OS and DFS rates were 30% and 29% in patients with high ESPN expression, and 54% and 43% in patients with low ESPN expression.

### Inhibition of endogenous ESPN suppresses ESCC cell proliferation

**Fig. 3:**
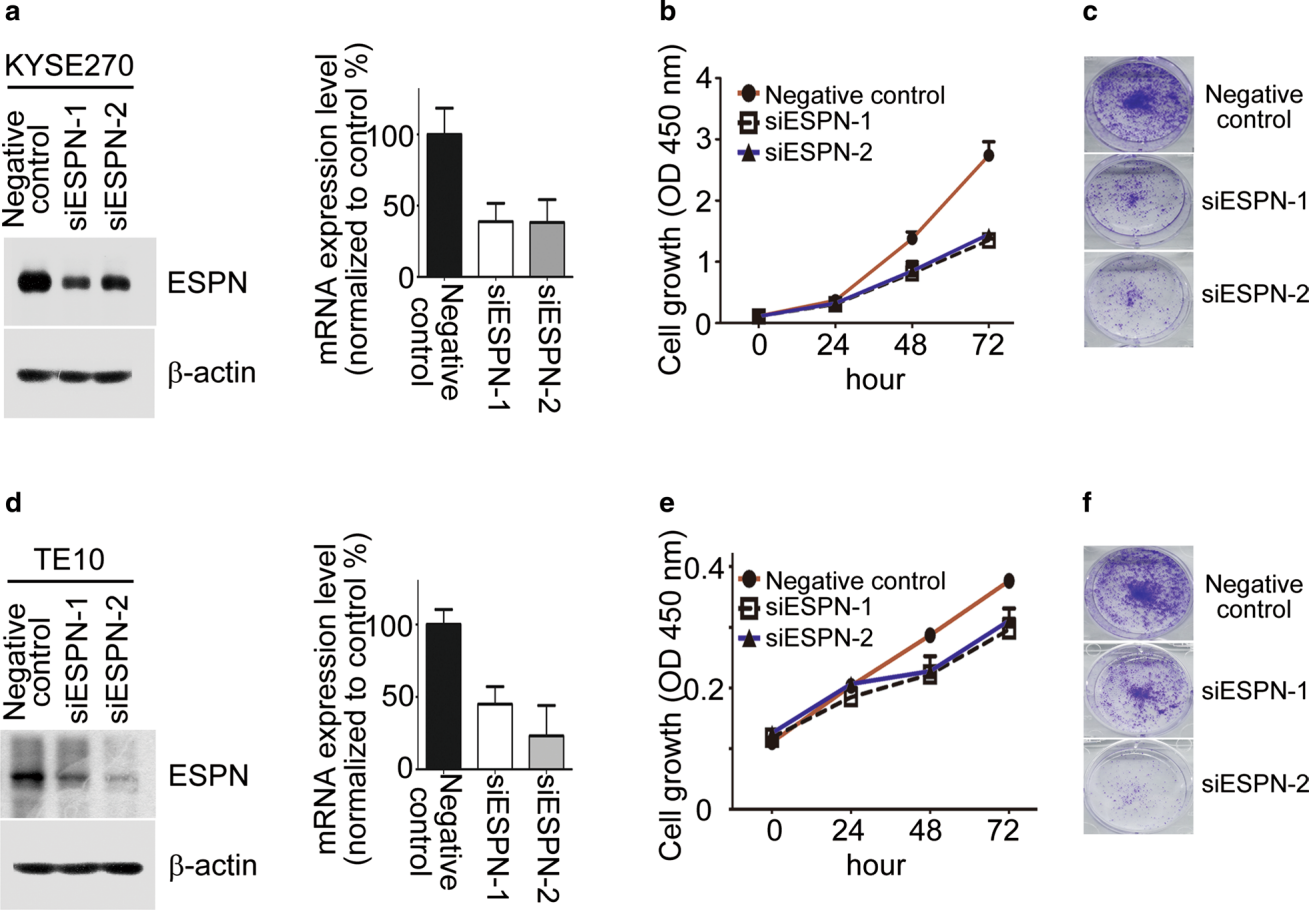
Knockdown of ESPN inhibited ESCC cells growth in vitro. **a**, **c** The endogenous expression of ESPN in KYSE270 and TE10 cell lines transfected with ESPN-mediated siRNAs and negative control were determined at mRNA and protein levels by qRT-PCR and Western blotting. **b**, **d** The cell viabilities and colony numbers of the siESPN-ESCC and negative control-ESCC cells were measured in different time points as indicated by MTT assay and colony formation assay. Data from three independent experiments are expressed as mean ± SD

The potential role of ESPN in ESCC progression remains unclear. Therefore, we carried out loss-of-function studies to determine the function of ESPN in ESCC cells. Two ESPN specific siRNAs were employed to interfere ESPN expression in KYSE270 and TE10. Both ESPN-mediated siRNAs were effectively decreased ESPN mRNA and protein expression levels (Fig. 3a, d). The MTT assays indicated that knockdown endogenous ESPN expression impaired cell proliferation in both ESCC cell lines (Fig. 3b, e, left panels). Furthermore, as demonstrated by the colony formation assay, the knockdown of ESPN in both ESCC cell lines decreased the colony numbers of cells, compared to the corresponding negative control group (Fig. 3c, f, right panels). These results demonstrated that interfering ESPN might inhibit ESCC growth by reducing cell proliferating rate.

#### ESPN inhibition did not alter the cell cycle progression, p21 and p27 expressions in ESCC cells

**Fig. 4:**
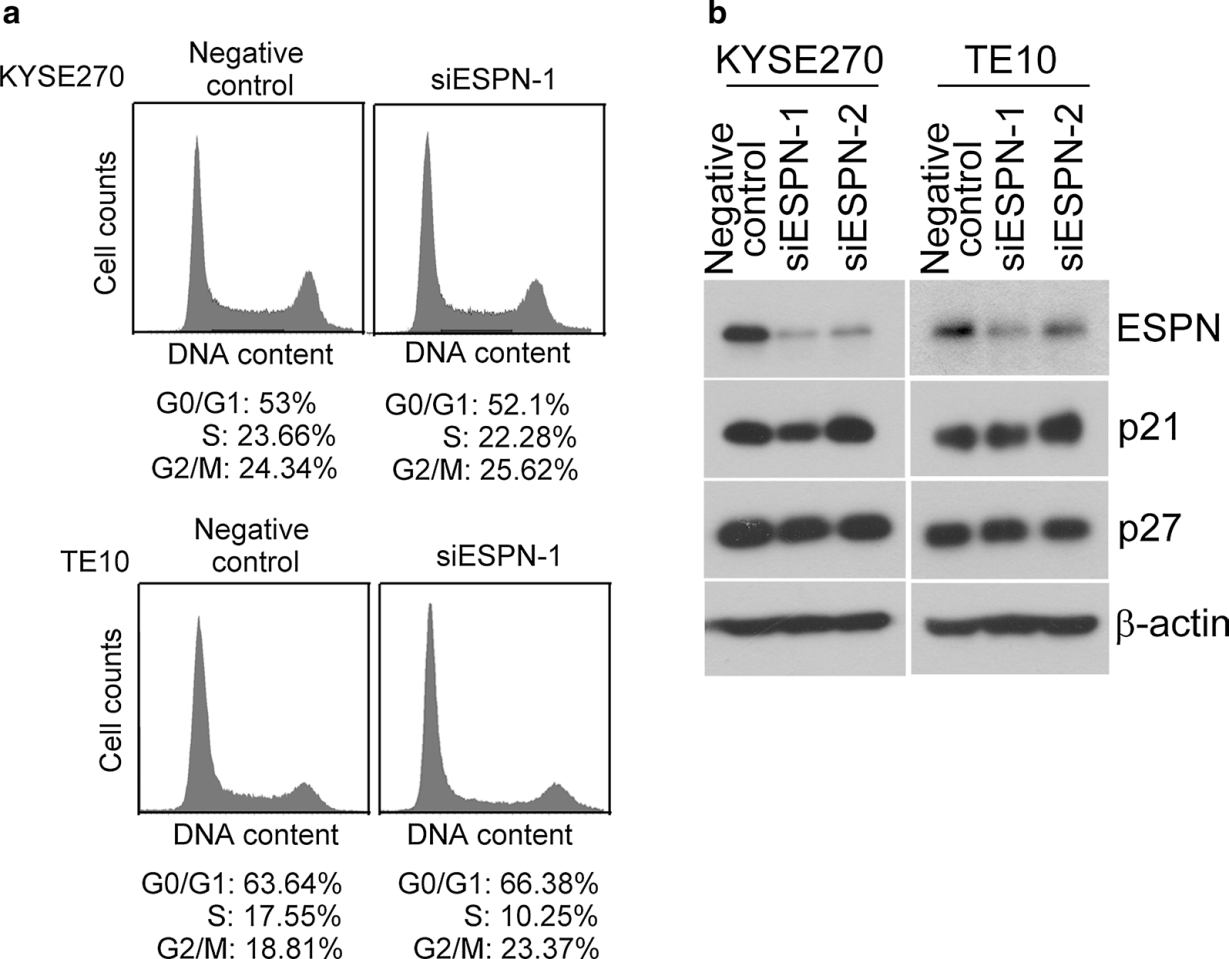
The cell cycle distribution, p21 and p27 expressions were determined in siESPN ESCC cells. **a** The cell cycle progression was analyzed by flow cytometry in negative control, ESPN-depleted KYSE270 and ESPN-depleted TE10 cells. **b** The protein expression levels of p21 and p27 were examined by Western blotting in negative control, ESPN-depleted KYSE270 and ESPN-depleted TE10 cells

Cell cycle distribution was carried out to investigate the effect of ESPN on cell cycle regulation. As shown in Fig. 4a, ESPN-depleted KYSE270 cells did not cause cell cycle alteration, compared to negative control group. Similar results also observed in siESPN-TE10 transfectants (Fig. 4a). Furthermore, using the same panels, the endogenous protein expression levels of p21 and p27 in siESPN-KYSE270 and siESPN-TE10 cells were not significantly higher than that of corresponding negative control cells (Fig. 4b).

## Discussion

Most of the patients with ESCC are diagnosed with advanced disease except for a few early stage ESCC found by screening in high‐risk populations such as patients with head and neck cancer history. Thus, the treatment outcome of the patients with ESCC receiving esophagectomy alone remains poor [2, 4, 5]. While the efficacy of preoperative therapy is relatively clear, the effect of postoperative adjuvant therapy remains controversial [14, 15]. Several studies of post-operative adjuvant therapy have been performed in an attempt to improve upon the survival rate achieved with esophagectomy alone, but the results are inconclusive. Therefore, identifying patients at high risk for recurrence after an esophagectomy who may actually benefit from postoperative adjuvant therapy is worthwhile and principal. In the present study, high ESPN expression was highly representative of biological aggressiveness and independently associated with poor disease-free survival. The 5-year disease-free survival rate was only 30% in patients with high ESPN expression, implying that ESPN status might be employed to select certain patients for adjuvant therapy after esophagectomy.

In our analysis, high ESPN expression had a negative prognostic impact on survival. However, we did not find significant correlations between ESPN expression and clinicopathologic parameters, such as TNM stage. Although there were more early stage diseases (69% versus 77%) in patients with low ESPN expression compared to patients with high ESPN expression, it did not reach significance (P = 0.21, Table 2). This discrepancy might be ascribed to the following reasons. First, we found IRS (immunoreactive score) 6 of ESPN expression to be the most prognostically effective cutoff point in survival analysis. Therefore, we used IRS 6 as cutoff point instead of IRS 4 in which we observed the significant correlation between ESPN expression with T classification and 7th AJCC Stage. Second, the cause might stem from the distribution of presenting stages in our cohort. In our institutes, many patients diagnosed with lymph node-positive ESCC gave up the attempt to undergo esophagectomy directly, and received neoadjuvant chemoradiotherapy first. Therefore, more than a half of ESCC (68%) in our series were lymph node-negative diseases. Third, there are still substantial differences in treatment outcome among ESCC patients with the same stage. Patients with the same pathological stage of ESCC that received the same surgical therapy may have distinct prognoses, and ESPN might be involved in intrinsic biologic aggressiveness which may lead to different treatment outcome in ESCC.

ESPN, an actin-filament binding protein regulates actin cytoskeleton, resulting in a special association with microvillar specializations of sensory cells [16]. ESPN can cross-links actin filaments to stabilize and elongate the actin bundles by preventing these bundles from disassembly and depolymerization [17]. In addition, ESPN expression or activity also involves in the elongation of filopodia, stereocilia in hair cells and microvilli in differentiated epithelial cells [18–20]. ESPN is able to organize F-actin bundles for promoting spinogenesis and spine maturation in mammalian cells [21, 22]. Altogether, these results implied that ESPN plays a role in actin bundles in the cells.

Recent studies in deaf mutant mice demonstrate that ESPN links function in the development and maintenance of normal stereocilia. ESPN mutation lost association with other actin-binding proteins or stereocilia-related proteins, such as, myosin XVa, myosin IIIa, Eps8, and whirlin results in abnormally short, long or fused stereocilia [17, 18, 23]. These results indicated that ESPN could maintain the organization and dimension of stereocilia which is essential for normal sound detection.

In human cancer, highly expressed ESPN in melanoma cells in mice and humans increased the metastatic ability of cancer cells through the regulation of lamellipodia formation [11]. Depletion of endogenous ESPN in melanoma cells causes cell growth reduction in vitro and in vivo; moreover, ESPN inhibition attest cell cycle at G1 phase via increasing p21 and p27 and decreasing Erk and AKT activities in melanoma cells [10, 11]. Recent study showed that miR-612 negatively regulated the expression of ESPN in melanoma cells. miR-612 targeted the 3′-UTR of ESPN mRNA to inhibit ESPN gene expression that suppressed melanoma cell growth, migration and invasion [7]. This finding demonstrated that the ESPN acts as a functional target gene of miR-612 in melanoma. Here, our study showed that high ESPN expression promoted cell growth and colony formation in ESCC cells. In contrast, inhibition endogenous ESPN by ESPN-mediated siRNA decreased cell growth ability. These results illustrated that ESPN expression involved in cell proliferation in ESCC cells. However, ESPN inhibition did not significantly alteration of cell cycle progression, p21 and p27 protein expressions in ESCC cells, indicating that ESPN regulated cell proliferation may through other mechanism in ESCC cells. We also determined if ESPN expression regulates ESCC cell migratory and invasive abilities. Our data indicated that the abilities of migration and invasion of cells were not altered in ESPN-depleted ESCC cells (data not shown). For detail mechanisms of ESPN-elicited cell proliferation in ESCC need to further investigation.

Our study has important limitations. First, the current study was a retrospective finding. Second, our observations were limited by the relatively small number of patients.

## Conclusions

This study presented that ESPN was upregulated in ESCC tissues compared with adjacent non-cancerous tissues. In addition, ESPN overexpression was positively correlated with overall survival and disease-free survival of ESCC patients, indicating ESPN may has significant meaning as a prognostic indicator for ESCC patients. To the best of our knowledge, this is the first study to illustrate that ESPN inhibition prevented the ESCC cell proliferation. Herein, ESPN could be a potential biomarker for the diagnosis of ESCC.
